# Effects of Quetiapine on Novelty‐Related Object Recognition Memory and Hippocampal BDNF Level in Sleep‐Deprived Rats

**DOI:** 10.1002/brb3.70226

**Published:** 2025-01-19

**Authors:** Öznur Özge Özcan, Burcu Çevreli, Emel Serdaroğlu Kaşıkçı, Mesut Karahan, Muhsin Konuk

**Affiliations:** ^1^ Electro‐Neurophysiology, Vocational School of Health Sciences Üsküdar University İstanbul Turkey; ^2^ Department of Physiology, Faculty of Medicine Üsküdar University İstanbul Turkey; ^3^ Department of Molecular Biology, Faculty of Engineering and Natural Sciences Üsküdar University İstanbul Turkey; ^4^ Medical Laboratory Techniques, Vocational School of Health Sciences Üsküdar University İstanbul Turkey

## Abstract

**Background:**

The underlying mechanism of quetiapine (QET) in treating cognitive impairment in sleep deprivation is unclear. The present study aimed to evaluate the effects of treatment with QET on novel object recognition and hippocampal (hippo) brain‐derived neurotrophic factor (BDNF) levels in rats submitted to 72 h sleep deprivation (SD).

**Materials and Methods:**

A total of 42 adult male Wistar albino rats were assigned into six experimental groups: non‐sleep‐deprived (NSD) control, short‐term control group (*n* = 7) received a single intraperitoneal (i.p.) injection 10 mg/kg QET of 1 mL saline (4 days) (NSD‐STQET), long‐term control group (*n* = 7) received single i.p. injection 10 mg/kg QET of 1 mL saline (30 days) (NSD‐LTQET); 72 h sleep‐deprived (SD) group, 72 h SD short‐term group received short‐term i.p. injection 10 mg/kg QET of either (*n* = 7) (SD‐STQET), and 72 h SD long‐term group received long‐term i.p. injection 10 mg/kg QET of either (*n* = 7) QET (SD‐LTQET). SD was performed using the modified multiple‐platform technique in a water tank for 72 h. Additionally, we aim to reveal the consequences of 72 h SD and QET effects on memory processes with hippo BDNF levels by testing rats in the novel object recognition (NOR) test and ELISA method.

**Results:**

Long‐term QET administration in healthy rats decreased NOR and BDNF protein expression in the hippocampus, as did 72 h SD. Long‐ and short‐term QET administration reversed SD effects, but only short‐term QET administration increased hippo BDNF.

**Conclusion:**

These results suggest that the beneficial effects of QET on SD may be partly related to the upregulation of recognition memory and neuroprotective proteins such as BDNF. However, long‐term QET treatment in the absence of a disease model may have the potential to negatively impact recognition memory and BDNF levels, which support synaptic plasticity and cognitive function.

## Graphical abstract

In our study, we evaluated the effects of short‐ and long‐term antipsychotic quetiapine use on learning and memory performance impaired by sleep deprivation, as well as without sleep deprivation, by behavioral analysis and measurement of BDNF levels in the hippocampus. We also measured anxiety levels by stool count.

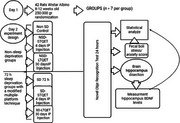

## 1 Introduction

In our modern world, there are diseases such as insomnia, narcolepsy, restless legs syndrome, and obstructive sleep apnea that are increasingly common (Morin and Jarrin 2022). Considering the population living in big cities, alcohol consumption, shift work, exposure to excessive light and noise, stress, anxiety, and various medical disorders are factors (Bhaskar, Hemavathy, and Prasad 2016). Sleep deprivation (SD) has been linked to a variety of negative outcomes, including higher anxiety, stress levels, and poor memory recognition (Havekes et al. 2016). During sleep, memory functions including spatial memory, recognition, long‐ and short‐term memories, and prospective memory are preserved and enhanced (Chen et al. 2023). SD negatively impacts the ability to form new memories. SD has been shown to affect memory significantly. As a result of SD, patients have varying degrees of impairment in spatial, visual, and working memory (Khan and Al‐Jahdali 2023). SD may reduce hippocampal activation during the encoding phase during wakefulness, resulting in impaired memory retrieval even after a night of recovery sleep (Havekes and Abel 2017). The hippocampus, in particular, is the most important cognitive function center, especially related to learning and memory (Heys et al. 2020; Sakai 2023).

Brain‐derived neurotrophic factor (BDNF) plays an important role in the pathophysiology of stress‐related mood disorders. The interaction of stress and sleep affects BDNF levels. SD can show significant changes in BDNF levels (Schmitt, Holsboer‐Trachsler, and Eckert 2016). Poor sleep quality is also associated with changes in BDNF concentration. Some authors suggest that in most cases, poor sleep quality increases stress and, therefore, susceptibility to depressive disorders, and that there is a relationship between sleep, depression, and BDNF levels. These changes in BDNF may contribute to memory impairment. BDNF promotes neuronal and synaptic growth and differentiation to regulate synaptic plasticity. If BDNF levels are reduced, neuroplasticity, essential for memory function, may be impaired (Dincheva, Lynch, and Lee 2016; Domitrovic Spudic et al. 2022; Leal, Bramham, and Duarte 2017; Lim et al. 2014). Limited studies reported that SD reduces hippocampal BDNF expression (Mahboubi et al. 2019; Mohammadipoor‐Ghasemabad et al. 2019; Rahmani, Rahmani, and Rezaei 2020; Saadati et al. 2014).

Increasing evidence suggests that REM SD impairs the formation and maintenance of learning and memory (Crowley, Bendor, and Javadi 2019; Fyfe 2020; Kim et al. 2022). Heckman et al. (2020) reported that 6 h SD impaired the acquisition, consolidation, and recall of object location memories in mice. Hajali et al. (2015) reported that 48 h SD by modified water tank method significantly disrupted short‐term memory and long‐term potentiation (LTP) using the Morris water maze (MWM) task, as well as hippo BDNF expression. In another study, Jiao et al. (2022) used novel object recognition (NOR) tasks to separately test the novelty‐related object location memory of mice induced by the modified water tank method for 24, 48, and 72 h of sleep deprivation. A total of 24 h SD enhanced NOR and hippocampal plasticity, 48 h SD had no effect, but 72 h SD severely impaired NOR and hippocampal synaptic plasticity.

The use of quetiapine (QET), a second‐generation antipsychotic drug, has increased sharply in recent years. Although it is approved by the US Food and Drug Administration (FDA) only for the treatment of schizophrenia, bipolar disorder (depression, acute mania, and maintenance), and major depressive disorder (as an add‐on medication) (Maan et al. 2023), QET is a common off‐label antipsychotic drug for treating insomnia. A recent meta‐analysis study suggested the use of QET in rational doses as an adjunctive drug for sleep disorders (Lin et al. 2023) but considering the type of receptor it affects and its affinity, research should be conducted on its healing or side effects (Modesto‐Lowe et al. 2021). Poddar et al. (2020) reported that 30 or 90 days of QET administration may help increase NOR memory performance and BDNF levels. Antipsychotic treatments, especially QET, may affect memory problems due to decreased hippo BDNF levels, which are particularly prevalent in cases of SD. To the best of our knowledge, no prior research has examined the likely impacts of QET on the NOR memory and hippocampus BDNF level in rats that have been sleep‐deprived (SD) for 72 h. The study described here aimed to evaluate the effects of QET, one of the most commonly prescribed second‐generation antipsychotics, on hippo BDNF levels, which are known to affect cognitive function and neuronal plasticity, particularly when used in the 72 h SD effect, where it is known to impair NOR memory. Like other second‐generation antipsychotics, QET is an antagonist of serotonin and dopamine receptors; however, its transient occupancy and rapid dissociation from postsynaptic dopamine receptors are thought to explain its favorable side effect profile, particularly its lower potential to produce extrapyramidal symptoms or hyperprolactinemia, compared with many other antipsychotics (Shettima et al. 2023). We aimed to investigate whether QET could reverse this effect, as recognition memory is a domain of cognition that is frequently impaired by the effects of SD (Ma et al. 2020; Raven et al. 2020). We also assessed the effects of QET specifically on hippo BDNF, given its important and well‐documented roles in synaptic plasticity and cognitive function.

## 2 Material and Methods

As experimental animals, 42 rats of the Wistar breed, male, 8–12 weeks old, 250–300 g in weight, with a maximum of 10 days between birth dates, were included in the study. All experiments were conducted according to the ethical guidelines in the Guidelines for the Care and Use of Laboratory Animals adopted by the US National Institutes of Health and published in 1996 and OECD guidelines no. 423. All animal experiments were performed with prior permission from the Animal Research Ethics Committee of Üsküdar University, İstanbul, Turkey. This study was approved by the Local Ethics Committee of Üsküdar University on January 20, 2023 the decision number of Ü.Ü‐HADYEK 2023–03.

All applications provided 12 h of light and 12 h of darkness (08:00–20:00, light), the temperature is 22°C ± 2°C, they have access to sufficient food and water, and the relative humidity is constant at 40%–45% humidity. It was conducted in the Experimental Research Unit of the Neuropsychopharmacology Application and Research Center (NPFUAM) at Üsküdar University.

**FIGURE 1:**
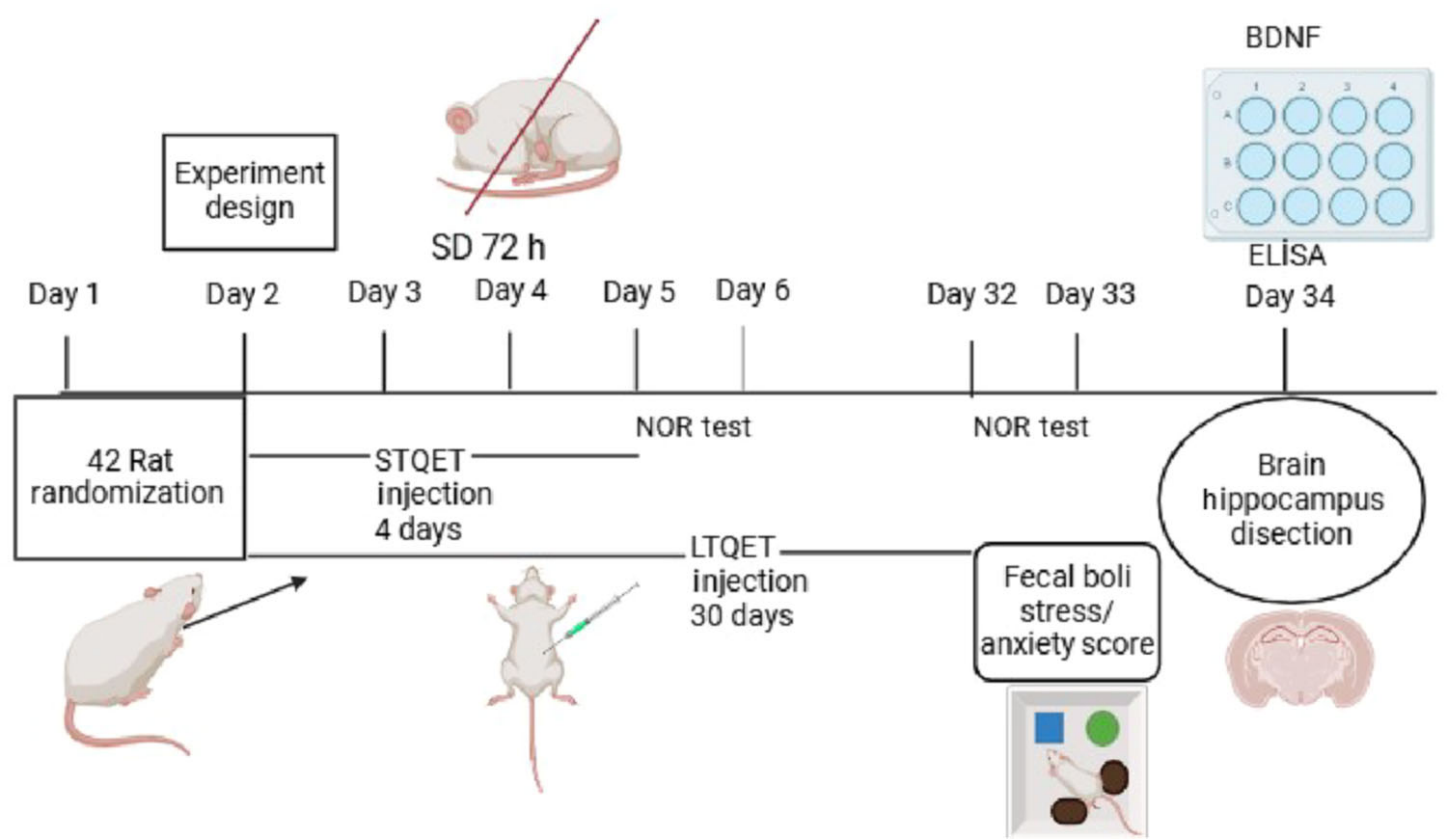
A timeline diagram for the experimental design.

A total of 42 adult male Wistar albino rats were assigned into six experimental groups: non‐sleep‐deprived (NSD) control, short‐term control group (*n* = 7) received a single intraperitoneal (i.p.) injection 10 mg/kg QET of 1 mL saline (4 days) (NSD‐STQET), long‐term control group (*n* = 7) received single i.p. injection 10 mg/kg QET of 1 mL saline (30 days) (NSD‐LTQET), 72 h SD group, 72 h SD short‐term group received short‐term i.p. injection 10 mg/kg QET of either (*n* = 7) (SD‐STQET), and 72 h SD long‐term group received long‐term i.p. injection 10 mg/kg QET of either (*n* = 7) QET (SD‐LTQET). All experimental designs are shown in Figure 1.

### 2.1 A Total of 72 h of Sleep Deprivation With a Modified Multiple‐Platform Technique

Rats were placed in stable social groups of seven within a water tank (dimensions: 125 cm × 43 cm × 45 cm) containing 14 small circular platforms, each approximately 6.5–7 cm in diameter. The tank was filled with water to 1 cm below the top of the platforms or grates, with a water height of 15 cm. After a total of 72 h of sleep deprivation, the rats were returned to their home cage for NOR assessments. Seven separate sleep deprivation experiments were conducted using different animals to achieve a final sample size of seven animals per group. The tank was equipped with grates to provide access to both food and water, similar to their home cages. All animals in each group were placed in the tank simultaneously. The modified multiple‐platform tank for sleep deprivation method was previously referenced in the study by Özcan et al. (2023).

### 2.2 Drug Administration

Pure QET (hemifumarate) was used in this study (Cayman Chemical, USA; CAS Number: 111974‐72‐2). The pure powder QET was injected via i.p. in 10 mg/kg/day dose dissolved in 1 mL saline. The dose chosen was by guidelines adapting human doses to animal doses. QET (10 mg/kg/day, intraperitoneally) dissolved in physiological saline was administered (He et al. 2020), and this dose/solvent was planned according to previous studies to avoid chronic drug‐induced deaths in experimental animals (Xiao et al. 2008; Zhou et al. 2020; Wang et al. 2015). According to the OECD, no. 407, injection volumes to be administered to animals at one time should not exceed 2 mL/100 g body weight, and the volume of all drug solutions injected into each rat in this study was 1 mL.

In the ST‐QET groups, 1 h after the first dose of QET administration, the SD model was applied with the multiple‐platform technique for 72 h, and daily drug doses were continued for 3 days throughout SD. The NOR test was performed 90 min after the end of the SD. In the LT‐QET groups, daily doses of drugs were injected intraperitoneally into rats. Following the injection on the 27th day, the animals were taken to 72 h SD. QET was continued for the last 4 days, and the injections were completed for 30 days. The NOR test was performed 90 min after the last injection. Fecal boli were counted for each mouse after completing the NOR to measure stress/anxiety levels following published literature (Ren et al. 2012; Swiergiel and Dunn 2006).

### 2.3 New Object Recognition Test

The test is used to measure attention, anxiety, working memory, and novelty preference in animals. NOR will be evaluated in a square open field using the new location recognition and new object recognition tasks, respectively. The apparatus is in dimensions of (40 cm × 40 cm × 40 cm), and the floor consists of a wooden box. The procedure consists of three phases: habituation, training, and testing. At each stage, the box was cleaned with 75% alcohol before the rats entered each cage. Animals are allowed to freely explore the new object recognition box for 5 min in the absence of objects during the habituation phase. In the training phase, two identical objects are placed (T1 and T2), and the animal is free to explore the objects. This time is recorded for each object (for 5 min), allowing the rats to explore these objects. The test phases are repeated after 24 h. An old object is replaced by a new object (N1), the other object remains the same (T2), and the sniffing and discrimination index is observed in 5 min. Thus, the animal's ability to perceive a familiar and new object in 24 h is examined (Goulart et al. 2010). Animals’ preference for new objects or new places is calculated using a discrimination index such as (time to discover new object − time to discover old object)/(time to discover old object + time to discover new object) (Bekci et al. 2024). The total time spent exploring both objects during the training session and the test session, as well as the number of stools, are also recorded.

### 2.4 Brain Samples

The animals were decapitated immediately after completing the NOR test. Rat hippocampi were dissected from the brains, rapidly frozen, and stored at −80°C until assayed. The samples of the hippocampus of the rats were homogenized in KCl KH_2_PO_4_ (12 mM KCl, 0.038 mM KH_2_PO_4_, pH = 7.4). The hippocampal region includes hippocampal formation and the parahippocampal region. The hippocampal formation is a C‐shaped structure located posteriorly in both hemispheres of the rat brain (Kjonigsen et al. 2015). In this study, the positioned region was dissected.

### 2.5 Measurement of BDNF Levels

For the analysis of neurotrophic factor, the dissected hippocampus was homogenized in phosphate‐buffered solution (PBS) with 1 mM phenylmethylsulfonyl fluoride (PMSF) and 1 mM ethylene glycol‐bis (2‐aminoethyl ether)‐N,N,N′,N′‐tetra acetic acid (EGTA). The homogenates were centrifuged at 10,000 g for 20 min, and the supernatants were collected to quantify the neurotrophic factor levels. BDNF levels in the hippocampus were evaluated by sandwich enzyme‐linked immunosorbent assay (ELISA) using commercial kits according to the manufacturer's instructions (BDNF levels were assessed with a kit from Sunred—201‐11‐0477 [Shanghai]). The ELISA reader captured the optical density.

### 2.6 Statistical Analysis

The data were evaluated using the GraphPad Prism v10.1.1 statistical software. We calculated sample sizes of minimum *n* = 7 animals per condition using the previous study of NOR task (Ameen‐Ali, Eacott, and Easton 2012), *α* = 0.05, and power = 0.80, to detect at least a 25% difference in mean values for treated and control samples using G*Power (Faul et al. 2007). A test for normality was performed to select the appropriate statistical method. All data are expressed as the mean ± SEM. The values were analyzed by one‐way ANOVA followed by post hoc analyses using Tukey's test to compare the groups (Prism, version 10.0; GraphPad Software Inc., San Diego, CA). *p* values less than 0.05 were considered statistically significant.

## 3 Results

### 3.1 Novel Object Recognition Test

**FIGURE 2:**
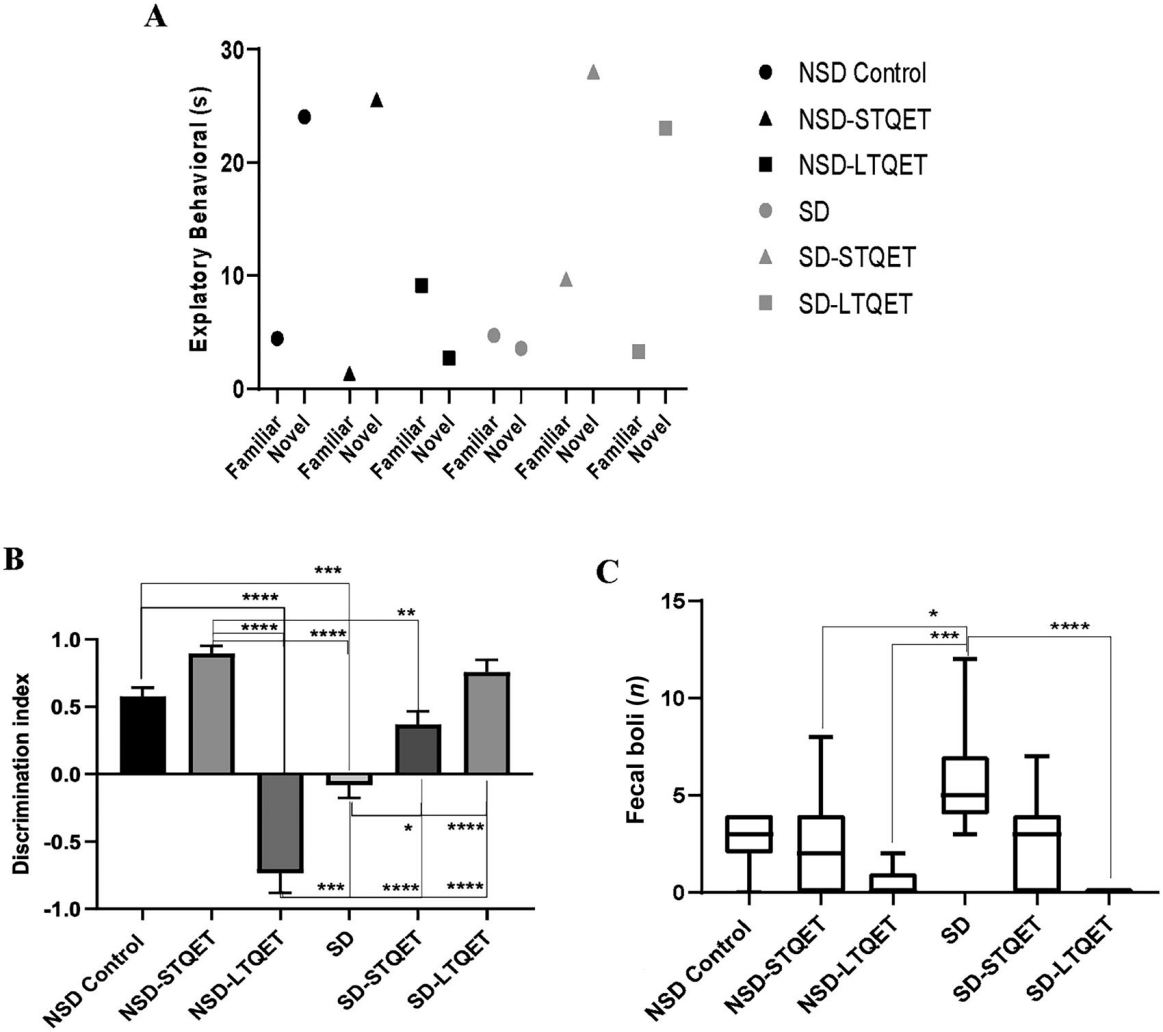
(A) Exploration times for familiar and novel objects, total exploration time (mean ± SEM) in the novel object recognition test session. (B) Discrimination index (mean ± SEM) for the test session. (C) Fecal boli was counted to evaluate stress/anxiety after the NOR test (*n* = 7 for each group); *p < 0.05, **p < 0.01, ***p < 0.001, and ****p < 0.0001.

Two‐way ANOVA results revealed no significant difference between the study groups for the exploratory behavior with the novel and familiar object (Figure 2A). Object exploration behavior was also analyzed by repeated measures ANOVA. Animals did not investigate the new object for a longer period, indicating an influence of the object, but it was not statistically significant (*p* = 0.0639, *F* (1, 5) = 5.621). No experimental groups explored the novel object significantly more than the familiar object (*p* = 0.6213, *F* (5, 5) = 0.7476).

The results of the NOR discrimination index are shown in Figure 2B. The differences between the groups in the NOR discrimination index of the 24‐h test phase were significant (*p *< 0.0001, *F* (5, 36) = 40.15).

According to univariate ANOVA analysis of the NOR discrimination index, 72 h SD had a main effect. A significant decrease was found in the SD group compared to the NSD control group (*p* = 0.0003, *t* = 6.841). Likewise, a significant decrease was found in NSD‐LTQET compared to NSD control (*p* < 0.0001, *t* = 13.62). In this case, 30‐day QET use had a more detrimental effect on visual memory than SD.

Interestingly, a significant decrease was found in the NSD‐LTQET group compared to the SD group (*p* = 0.0004, *t* = 6.781). The same results were found in the NSD‐STQET group that significant decrease was found in the SD‐STQET group (*p* = 0.0054, *t* = 5.476). Short‐term QET use in the presence of SD affects visual memory more negatively than in the absence of SD.

The SD‐STQET (*p* < 0.0001, *t* = 11.46) and SD‐LTQET (*p* < 0.0001, *t* = 15.49) groups showed a significant increase compared to the NSD‐LTQET group. Long‐term QET without SD significantly impaired the NOR discrimination index compared to long‐term and short‐term QET in the presence of sleep deprivation.

The NSD‐LTQET group showed a significant decrease compared with the NSD‐STQET group (*p* < 0.0001, *t* = 11.46). Short‐term QET use has no detrimental effect on visual memory compared to long‐term QET use.

The number of fecal boli was 2.571 ± 2.286 for group NSD, 2.286 ± 5.429 for group NSD‐STQET, 0.4286 ± 0.157 for group NSD‐LTQET, 5.857 ± 8.574 for group SD, 2.857 ± 4.890 for group SD‐STQET, and 0 ± 0 for group SD‐LTQET. The results of fecal boli are shown in Figure 2C. The differences between the groups for the fecal boli after the NOR test were significant (*p* = 0.0001, *F* (5, 36) = 7.025). Fecal boli were counted to measure stress‐/anxiety‐like behavior after NOR test completion, and only the SD group had significantly higher fecal boli compared to the NSD‐STQET group (*p* = 0.0312, *t* = 4.531), NSD‐LTQET (*p* = 0.0003, *t* = 6.887), and SD‐LTQET group (*p *< 0.0001, *t* = 7.431).

### 3.2 BDNF Levels

**FIGURE 3:**
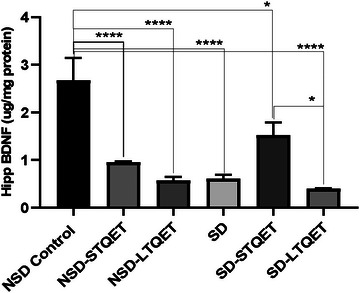
Effects of QET administration on BDNF levels in the hippocampus of animals subjected to SD in the hippocampus (hipp) (*n* = 7 per group). Data were analyzed by one‐way ANOVA, followed by Tukey's test when *F* was significant. Values are expressed as the mean ± SEM; *p < 0.05, **** *p *< 0.0001.

In the current study, we took brain tissue samples from all groups for the ELISA method to understand the levels of BDNF in the hippocampus. When BDNF is detected in the hippocampal region isolated from brain tissue, this circumstance becomes even more significant because it enables us to conduct critical assessments about visual memory practices. A total of 72 h SD decreased the levels of BDNF in the hippocampus of rats. Figure 3 shows the effects of short‐ and long‐term QET administration on the levels of BDNF in the hippocampus of rats submitted to 72 h SD.

The amount of hippo BDNF (see in Table 1) was 2.671 ± 1.686 µg/mg for group NSD, 0.9541 ± 0.029 µg/mg for group NSD‐STQET, 0.5680 ± 0.057 µg/mg for group NSD‐LTQET, 0.6050 ± 0.074 µg/mg for group SD, 1.526 ± 0.390 µg/mg for group SD‐STQET, and 0.3951 ± 0.004 µg/mg for group SD‐LTQET.

**TABLE 1:** BDNF levels in the hippocampus as measured by ELISA. For more information, see Figure 3.

Groups BDNF (µg/mg)	
NSD control	SD
2.671 ± 1.686^b^	0.6050 ± 0.074^a^
NSD‐STQET	SD‐STQET
0.9541 ± 0.029^a^	1.526 ± 0.390^a^
NSD‐LTQET	SD‐LTQET
0.5680 ± 0.057^a^	0.3951 ± 0.004^a,b^

*Note*: Data were analyzed by one‐way ANOVA, followed by Tukey's test when *F* was significant. Values are expressed as the mean ± SEM; *n* = 7 rats in each group.

^a^ Significant compared to the NSD control group.

^b^ Significant compared to the SD‐STQET group.

In one‐way ANOVA, the differences between the groups in the BDNF levels were significant (*p *< 0.0001, *F* (5, 36) = 14.42). The average mean BDNF levels significantly decreased in the SD group (*p *< 0.0001, *T* = 9.129) compared to the NSD control group. A total of 72 h SD decreased almost three times the levels of BDNF in the hippocampus of rats. Likewise, BDNF levels significantly decreased in NSD‐STQET (*p *< 0.0001, *t* = 7.586) and NSD‐LTQET (*p *< 0.0001, *T* = 9.292). Short‐ and long‐term treatment with QET (10 mg/kg/day) also resulted in decreased BDNF levels in the hippocampal region. Interestingly, BDNF levels were increased in the SD‐STQET group compared to the SD group but not significantly (*p* = 0.0675, *t* = 4.071). However, this increase was significant compared to the SD‐LTQET group (*p* = 0.013, *t* = 4.999). Accordingly, although an increase in BDNF levels was observed with short‐term QET when exposed to SD, a decrease in BDNF levels can be seen when used long‐term. Short‐ (*p* = 0.012, *t* = 5.057) and long‐term QET use (*p *< 0.0001, *t* = 10.06) in SD exposure significantly decreased hippo BDNF levels compared to NSD control.

## 4 Discussion

This study investigated the effects of long‐ and short‐term QET use with 72 s SD on NOR test and hippo BDNF levels. The NOR test is used to assess memory for object identity (Lueptow 2017). Previous studies have shown that SD can induce object recognition memory impairment in animals. In this study, we showed that 72 h SD decreased BDNF levels in the total hippocampus. Moreover, at the behavioral level, our results also revealed that 72 h SD harmed NOR‐dependent cognitive performance within a certain time frame. Long‐term and short‐term QET use supported the NOR‐dependent cognitive performance enhancement caused by 72 h SD and suggested that this improvement may be related to the development of neurotrophic factors. However, interestingly, we also showed that long‐term QET use in healthy rats impaired NOR‐dependent cognitive performance and at the same time decreased BDNF levels in the total hippocampus. Long‐term QET administration resulted in the same cognitive and neurotrophic effects as 72 h SD administration. Jiao et al. (2022) reported that 72 h SD impaired novel object recognition, caused severe loss of hippocampal dendritic spines, and decreased GluA1 levels. This in turn reduced neuroplasticity in the hippocampus, negatively affecting LTP (Jiao et al. 2022). This indicates that there may be a negative relationship between objection recognition memory and hippo BDNF in the 72 h SD rats. Our results were also related to decreased BDNF levels and NOR disruption in the hippocampus. Parallel results were found in our study regarding SD at different times. Cordeira et al. (2018) demonstrated that 12 h SD significantly reduced novel object preference compared to the control group. This indicates that sleep deprivation impaired object recognition memory. Also, Palchykova et al. (2006) reported that during the test phase, 0–6 SD mice were unable to discriminate between a single novel and two familiar objects, but the 7–12 SD group and two control groups explored the novel object significantly longer than the two familiar objects. In both adolescent and adult mice, 72 h SD impaired memory in a NOR test, but an increase in the density of excitatory synapses in the granule cells of the dentate gyrus in the hippocampus of adolescent mice was reported (Tuan and Lee 2019). In Ishikawa et al. (2014), study 4 h SD impaired object‐place recognition but not NOR. A total of 8 h SD did not significantly impair either. Exposure to SD at different times perhaps suggests that sleep selectively promotes hippocampal‐dependent memory consolidation and that this effect is limited to 4–12 h after learning. Literature shows controversial results due to the effects of many factors related to SD. Gender, age, duration, and models of SD have controversial effects on NOR. In our study, adult male rats and 72 h SD with a modified water tank were used, referenced from our previous study (Özcan et al. 2023).

The novel object discrimination indexes for long‐term QET administration in the control group and 72 h SD were significantly lower, respectively. This means that they did not spend significantly more time with the novel object compared to the familiar object. However, there was no significant difference between the study groups for the exploratory behavior with the novel and familiar objects. Our results emphasized the adverse effects of 72 h SD on NOR despite many factors. Apart from visual memory, novelty seeking is a personality trait characterized by the tendency to seek out new experiences that evoke strong emotions (Wingo et al. 2016). This behavioral construct includes novelty preference, reward dependence, and risk‐taking. On the other hand, novelty‐seeking behavior is measured by calculating the time the rodent spends exploring the novel object compared to the time it spends on the familiar object, thus focusing on the preference behavior aspects of novelty (Antunes and Biala 2012; Wooden et al. 2021). According to our results, the 72 h SD and long‐term QET control rat groups spent more time with the familiar object than with the novel object but not significantly.

To our knowledge, the effect of QET on SD‐induced object recognition memory impairment and hippo BDNF level is not known. The possible therapeutic effect of short‐ and long‐term QET on the 72 SD‐induced recognition memory impairment and hippo BDNF level was evaluated. The antipsychotic mechanism of QET is related to its potent inhibition of the serotonin 5HT2A receptor and its low affinity for the dopamine D2 receptor, unlike typical antipsychotics (Chamera et al. 2023). Treatment with chronic QET recovered memory impairment in the amyloid precursor protein (APP)/presenilin‐1 (PS‐1) double transgenic (TG) mouse model of Alzheimer's disease (He et al. 2009). In another study, 16 days of acute QET (10 mg/kg/day, i.p.) reduced hippocampal oxidative stress and had beneficial effects on object recognition memory in phencyclidine rats (He et al. 2018). Mani and Alshammeri (2023) reported that 30 days of QET administration (10 or 20 mg/kg, p.o.) increased the exploration time of the novel object and the discrimination ability of the objects in NOR in DOX‐induced cognitive deficits. Additionally, 28 days of QET administration (10 mg/kg/day, i.p.) reversed methamphetamine‐induced recognition memory impairment in NOR (He et al. 2006). There may be some factors linking the reversing effects of QET on the neurotoxicity and memory impairment induced by 72 h SD.

Among possible factors, a neuroprotective protein, BDNF, is a strong candidate. According to the results of the studies, in disease states that generally negatively affect BDNF levels in the hippocampus, the ability of QET to increase these levels is remarkable. QET prevented the reduction of BDNF levels induced by immobilization stress in rat hippocampus (Xu et al. 2002). The study demonstrated that QET enhanced BDNF mRNA expression in the dentate gyrus of the rat hippocampus, even in baseline conditions. This observation aligns with a recent study that examined BDNF mRNA levels following a 28‐day treatment with clozapine and olanzapine, both of which, like QET, are classified as atypical antipsychotics (Bai et al. 2003). In a TG Alzheimer's disease model, QET elevated BDNF levels in the basolateral amygdala and hippocampus of mice (Tempier et al. 2013). In our study, 30 and 4 days of administration of QET (10 mg/kg/day, i.p.) reversed the destructive effect of 72 h SD on object recognition memory, but only short‐term QET administration increased hippo BDNF levels significantly. Interestingly, in another study, the chronic administration (21 days) of QET (10 mg/kg) significantly attenuated the decreased BDNF mRNA expression in both hippocampi but significantly increased the BDNF mRNA expression in the dentate gyrus of rats (Park et al. 2006). Poddar et al. (2020) reported that chronic QET (oral, 25 mg/kg/day, 90 days) treatment has the potential to negatively impact recognition memory (NOR test) and neurotrophin‐related signaling molecules (BDNF levels) in adult rats that support synaptic plasticity and cognitive function. In our study, although NOR was not affected by short‐term (4 days, 10 mg/kg/day, i.p.) QET use, long‐term use of 30 days impaired NOR. Short‐ and long‐term QET use significantly reduced BDNF levels in the hippocampal region compared to the control group. In particular, long‐term QET use harmed BDNF levels as much as the effect of 72 h SD. Despite different doses and routes of administration of QET on different days, the results were parallel. The effects of antipsychotics on BDNF expression are controversial. The effect of QET intake is quite limited in the literature. More research is needed on hippo BDNF in learning, memory, and cognitive deficits related to SD with more behavioral methods. Therefore, QET may benefit the regulation of 72 h SD‐induced NOR‐dependent BDNF levels with potential neuroprotective effects. In addition, the limitation of our current study is the lack of direct morphological evidence demonstrating the effects of QET and SD on other neurotrophic factors. Future studies are also recommended to elucidate the role of many other neurotrophic factor levels, myelination, and BDNF in oligodendrocytes in the therapeutic effect of QET in SD‐induced cognitive impairments.

Fecal boli counting has been used in previous studies mostly in open‐field tests (Haider et al. 2014; Sadegzadeh et al. 2020; Seibenhener and Wooten 2015). Platano et al. (2008) used fecal boli amount to measure anxiety levels in the NOR test. We also measured defecation or fecal boli counting after the NOR test. In the 72 h SD group, the amount of fecal bolus was higher in short‐ and long‐term QET control groups. A total of 72 h SD group had significantly higher fecal boli that demonstrated higher stress‐/anxiety‐like behavior compared to long‐term QET administration groups. Long‐term use of QET in rats exposed to SD for 72 h resulted in a significant reduction in fecal bolus amounts. Long‐term use of QET may be beneficial in managing anxiety and stress due to sleep deprivation.

## 5 Conclusion

We evaluated long‐term memory function (24 h) by NOR test, where decreased recognition memory was observed in the long‐term QET administration control group and 72 h SD group. However, no significant difference has been observed in exploratory behavior in all groups. Although the NSD control group spent more time with the novel object than the familiar object, the difference was not statistically significant. These results suggest that QET may improve 72 h SD‐mediated cognitive impairment and promote neuroplasticity through the upregulation of neurotrophic factors. Additionally, long‐term QET administration induced the same down regulation of neurotrophic factors and cognitive impairment as 72 h SD in healthy rats.

## Author Contributions

**Öznur Özge Özcan**: methodology, data curation, validation, writing–original draft, conceptualization, writing–review and editing, software. **Burcu Çevreli**: methodology, conceptualization, software, data curation, validation, investigation, writing–review and editing, supervision. **Emel Serdaroğlu Kaşıkçı**: methodology, conceptualization, supervision. **Mesut Karahan**: conceptualization, investigation, funding acquisition, writing–review and editing, methodology, writing–original draft, supervision, project administration. **Muhsin Konuk**: writing–original draft, writing–review and editing, methodology, data curation, funding acquisition.

## Conflicts of Interest

The authors declare no conflicts of interest.

### Peer Review

The peer review history for this article is available at https://publons.com/publon/10.1002/brb3.70226.

## Data Availability

The data that support the findings of this study are available on request from the corresponding author. The data are not publicly available due to privacy or ethical restrictions.
